# Hypoxia and Acidification Have Additive and Synergistic Negative Effects on the Growth, Survival, and Metamorphosis of Early Life Stage Bivalves

**DOI:** 10.1371/journal.pone.0083648

**Published:** 2014-01-08

**Authors:** Christopher J. Gobler, Elizabeth L. DePasquale, Andrew W. Griffith, Hannes Baumann

**Affiliations:** School of Marine and Atmospheric Sciences, Stony Brook University, Southampton, New York, United States of America; University of Gothenburg, Sweden

## Abstract

Low oxygen zones in coastal and open ocean ecosystems have expanded in recent decades, a trend that will accelerate with climatic warming. There is growing recognition that low oxygen regions of the ocean are also acidified, a condition that will intensify with rising levels of atmospheric CO_2_. Presently, however, the concurrent effects of low oxygen and acidification on marine organisms are largely unknown, as most prior studies of marine hypoxia have not considered pH levels. We experimentally assessed the consequences of hypoxic and acidified water for early life stage bivalves (bay scallops, *Argopecten irradians*, and hard clams, *Mercenaria mercenaria*), marine organisms of significant economic and ecological value and sensitive to climate change. In larval scallops, experimental and naturally-occurring acidification (pH, total scale  = 7.4–7.6) reduced survivorship (by >50%), low oxygen (30–50 µM) inhibited growth and metamorphosis (by >50%), and the two stressors combined produced additively negative outcomes. In early life stage clams, however, hypoxic waters led to 30% higher mortality, while acidified waters significantly reduced growth (by 60%). Later stage clams were resistant to hypoxia or acidification separately but experienced significantly (40%) reduced growth rates when exposed to both conditions simultaneously. Collectively, these findings demonstrate that the consequences of low oxygen and acidification for early life stage bivalves, and likely other marine organisms, are more severe than would be predicted by either individual stressor and thus must be considered together when assessing how ocean animals respond to these conditions both today and under future climate change scenarios.

## Introduction

Low oxygen zones are ubiquitous features in coastal and open ocean environments. Within coastal zones, the excessive delivery of nutrients from agriculture and urban centers stimulates algal productivity, and the subsequent microbial degradation of this organic matter reduces oxygen levels, contributing towards hypoxia [1]. Hypoxic regions of coastal zones have expanded in recent decades [1], [2] to the detriment of resident organisms and fisheries [2]–[6]. In the open ocean, oxygen minimum zones are persistent features of subsurface waters with rates of microbial oxygen consumption that exceed oxygen ventilation rates and are expanding due to ocean warming [7]–[9]. A less frequently considered consequence of microbial respiration is the production of CO_2_ and the resultant reduction in pH through the formation and dissociation of carbonic acid. Through the process of respiration, levels of CO_2_ and O_2_ are stoichiometrically linked in marine ecosystems, being subsequently altered by differences in gas exchange and chemical equilibria. Recent studies have indeed shown a close correspondence between low oxygen and acidification in many coastal and open ocean systems [10]–[13], with pCO_2_ levels in low oxygen zones often of a magnitude greater than predicted for surface oceans later this century (>1,000 µatm)[10], [11], [14]. The continued combustion of fossil fuels and subsequent warming of the planet are expected to expand low oxygen and acidified regions in the world's oceans this century [15]–[17].

**Figure 1:**
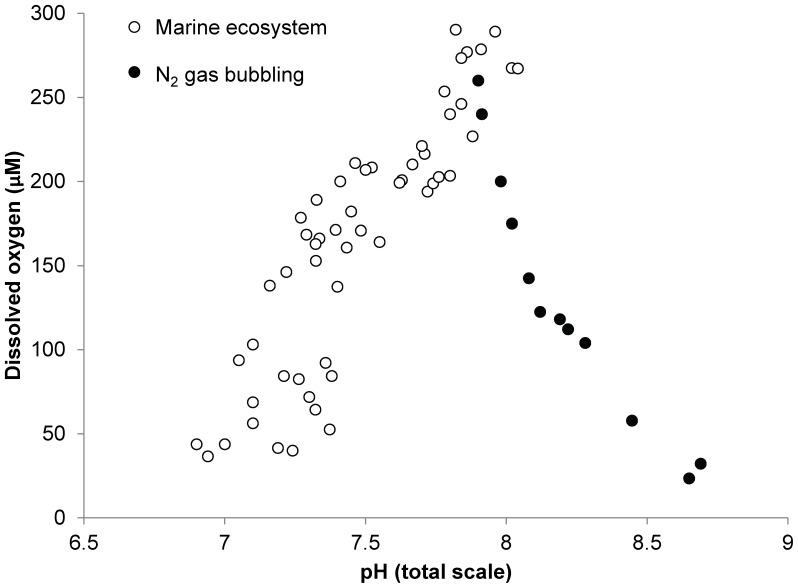
Changes in pH_T_ (total scale) and dissolved oxygen obtained via bubbling estuarine seawater (salinity  = 30) with N_2_ gas (black circles) and found naturally (white circles) in the Forge River, NY, USA, during summer 2012.

Despite the common co-occurrence of hypoxia and acidification in marine systems, their concurrent effects on ocean life are poorly understood. Acidification negatively impacts an array of marine organisms [17]–[19]. Oxygen is required to sustain aerobic life, and the thresholds at which low dissolved oxygen causes mortality and/or harm have been established for many marine organisms [2]–[6]. However, the large majority of laboratory studies assessing the effects of low oxygen on marine organisms have manipulated oxygen levels by administering nitrogen gas [e.g. 21]–[27], a process that reduces both oxygen and pCO_2_ levels and thus significantly increases, rather than decreases seawater pH (Figure 1). Hence, the majority of hypoxia studies performed to date have created a laboratory condition (low oxygen, low CO_2_, high pH) that does not reflect the actual physiological challenges to marine life in low oxygen zones (Figure 1).

**Figure 2:**
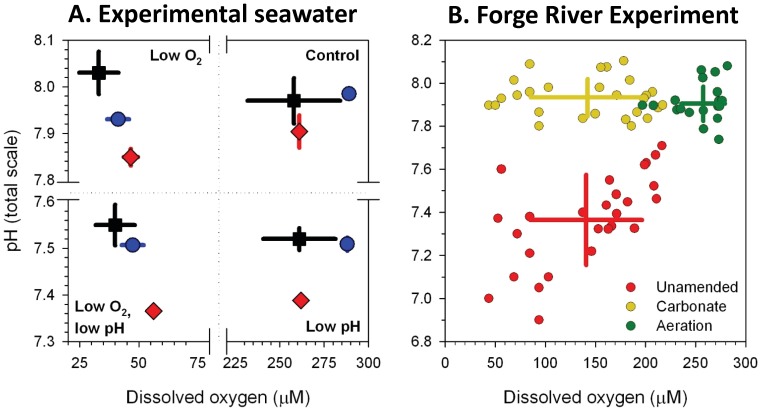
Levels of dissolved oxygen and pH_T_ during experiments. **A**. Experiments delivering N_2_ gas, CO_2_ gas, and air to seawater with *A. irradians* larvae (black squares), two month old *M. mercenaria* (red diamonds), or four month old *M. mercenaria* (blue circles) as presented in Figures 2, 4 and 5. Points are means ±1 S.D. **B**. Experiment with water from the Forge River, NY, USA, and *A. irradians* larvae left unamended (red), amended with sodium carbonate (yellow), or aerated (green). Points are means of measurements during individual days of the experiment. Crosses present the mean ±S.D. for the entire experiment.

Here, we examine the concurrent impacts of acidification and low oxygen on the early life stages of two calcifying bivalves: bay scallops, *Argopecten irradians*, and hard clams, *Mercenaria mercenaria*. As foundational species within coastal ecosystems [28], calcifying bivalves such as those studied here are of substantial value to man: mollusk harvests approach one billion USD annually in the US alone [29] with ecosystem services far exceeding that value [30]. The negative impacts of climate change on the early life history stages such as those studied here may promote population bottlenecks in bivalve populations [31]. While early life stage clams and scallops are sensitive to ocean acidification [19], [20], the effects of low oxygen on these organisms under acidified conditions have yet to be examined. For this study, we specifically hypothesized that early life growth, development (metamorphosis), and survival of these bivalves would be differently affected by hypoxic vs. acidified conditions, and that concurrent hypoxia and acidification would have additive negative effects. During experiments, levels of dissolved oxygen, pCO_2_, and pH_T_ (pH measured on the total scale) in seawater were achieved by mixing air, CO_2_, and N_2_ gases, as well as by amending naturally hypoxic and acidified coastal water from the Forge River Estuary, NY, USA, with aeration and sodium carbonate (Figure 2; Tables S1, S2, S3, S4).

## Methods

Two types of experiments are presented in this manuscript. The first experiments involved the exposure of early life stage bivalves (larvae and first year juveniles) to normal and low pH/low dissolved oxygen conditions achieved via the delivery of varying mixtures of CO_2_ gas, N_2_ gas, and air. The second experiment involved the exposure of larval bivalves to water low in low pH and low dissolved oxygen obtained from a hypoxic and acidified ecosystem that was subsequently manipulated via aeration and the addition of sodium carbonate. The first experiment was performed on larval stage bay scallops (*A. irradians*) and two different stages of juvenile hard clams (*M. mercenaria*), whereas the second experiment was performed on larval stage *A. irradians*. For all experiments, replicate (*n* = 4) experimental vessels with early life stage bivalves (described below) were maintained at 24±1°C using commercially available aquarium heaters and chillers (Aquatic Eco-systems, Inc., Florida, USA). This temperature is optimal for growth and survival of early life stages of the two species used here [32], [33].

### Manipulating pH and dissolved oxygen levels with tanked gas

A series of gas proportionators (Cole Parmer Flowmeter system, multitube frame) were used to deliver CO_2_ gas, N_2_ gas, and air to seawater at different rates to maintain conditions that were normoxic with a normal pH (air only), hypoxic with a normal pH (tanked N_2_ gas pre-mixed with ∼390 µatm CO_2_ gas), normoxic but acidified (tanked 5% CO_2_ gas only), and hypoxic and acidified (tanked N_2_ gas, tanked 5% CO_2_ gas, and air; Figure 2A). The gas proportionators delivered these gases to experimental vessels filled with 0.2 *µ*m filtered seawater from eastern Shinnecock Bay, New York, USA, to yield the levels of pH and dissolved oxygen desired for experiments using volumetric flow rates that turned over the volume of experimental vessels >100 times daily preventing equilibration with atmospheric CO_2_ and oxygen. For experiments with larvae, the gas mixtures from the proportionator system were continuously delivered to the bottom of 1 L high density polyethylene beakers with polycarbonate lids whereas 10 L polyethylene vessels with polyethylene lids were used for experiments with juvenile bivalves. Vessels were bubbled for least 24 h prior to commencing experiments. Dissolved oxygen levels were quantified with a Clark-type electrode YSI 5100 oxygen meter, which provided an analytical precision of ±4% and was calibrated daily. Dissolved oxygen measurements made with this probe were nearly identical to, and never significantly different from, those obtained via Winkler titrations [34]. To quantify precise levels of total dissolved inorganic carbon attained in experimental treatments, water samples were analyzed during experiments with an EGM-4, Environmental Gas Analyzer® (PP Systems) system that quantified total dissolved inorganic carbon levels after separating the gas phase from seawater using a Liqui-Cel® Membrane (Membrana). This instrument provided a methodological precision ±3% for replicated measurements of total dissolved inorganic carbon and provided full recovery (104±5%) of Dr. Andrew Dickson's (University of California San Diego, Scripps Institution of Oceanography) certified reference material for total inorganic carbon in seawater (Batch 117 = DIC 2009.99 µmol dissolved inorganic carbon kg seawater^−1^). Levels of CO_2_ were calculated based on measured levels of total inorganic carbon, pH_T_ (mol kg seawater^−1^), temperature, salinity, phosphate, silicate, and first and second dissociation constants of carbonic acid in seawater [35] using the program CO2SYS (http://cdiac.ornl.gov/ftp/co2sys/). Daily measurements of pH used a Honeywell Durafet Ion Sensitive Field Effect Transistor (ISFET)-based pH sensor calibrated with Dr. Dickson's seawater pH standard [36], which indicated that experimental vessels maintained target pH levels throughout experiments (<0.1% relative standard deviation within treatments). Spectrophotometric measurements of pH made using *m*-cresol purple as described by Dickson *et al.* [37] were nearly identical to, and never significantly different from, those obtained with the Durafet pH sensor.

### Experiments with naturally hypoxic and acidified water

The Forge River estuary (40°47′71″N, 72°49′85″W) is a small (3 km^2^), shallow (2–4 m), microtidal (0.7 m) system exchanging with Moriches Bay, a part of Long Island's south shore lagoonal system in NY, USA [38]. The Forge River receives excessive nutrient loads from dense residential populations and duck farms and experiences hypoxia during summer months [38]. While the two bivalves studied here (*M. mercenaria, A. irradians*) can be found along the entire eastern shore of North America including estuaries that exchange with and surround the Forge River such as such as Moriches Bay and the Peconic Estuary (see below), benthic surveys reveal these organism do not persist in the Forge River (C. Gobler, pers. obs.). For experiments, water from the Forge River was collected ten times during July and August of 2012 from the Town of Brookhaven (NY, USA) public dock at dawn from a depth of ∼2 m with a horizontal Van Dorn bottle. Water was transferred without bubbling to 20 L carboys and transported to the Stony Brook-Southampton Marine Sciences Center. Specific permission was not needed for this activity since this was a public location and seawater collection is not regulated by any municipal government. The pH and dissolved oxygen of the water was measured as described above and the salinity of the water was determined using a YSI© 556 sonde. Water from carboys was transferred by gravity, without bubbling to replicate (n = 4) 1 L high density polyethylene beakers with polycarbonate lids for experiments. Experimental water was manipulated with sodium carbonate or aeration to contrast changes in pH (carbonate addition only) to changes in dissolved oxygen and pH concurrently (aeration). Specifically, replicate vessels (n = 4) were left unamended as a control, aerated at 50 ml min^−1^ with ambient air, or administered 26 µM sodium carbonate per 0.1 pH unit difference between the experimental water and a pH value of 8.0. Prior to manipulation, water was always low in oxygen (15–90 µM) and pH (7.1–7.7). The addition of sodium carbonate brought pH, carbonate ion, and aragonite saturation states to values nearly identical to the aeration treatment but did not alter dissolved oxygen while bubbling typically brought pH to ∼8 and dissolved oxygen to ∼250 µM (Figure 2B). Dissolved oxygen, pH, and precise carbonate chemistry levels were monitored as described above and are reported in the results section and Table S2. Water was exchanged ∼ every three days, and levels of dissolved oxygen and pH generally rose between water changes.

### Larval experiments

Larval bay scallops (*A. irradians*) were obtained from broodstock collected from the eastern Peconic Estuary that experiences levels of pH, dissolved oxygen, and salinity common for a meso-trophic estuary (pH_NBS_ range: 7.8–8.2, mean ±S.D. = 8.0±0.16; dissolved oxygen: 170–380 µM, mean  = 250±60 µM; salinity  = 24.6–30.8, mean  = 28.1±1.34; [39]). Broodstock were collected by the East Hampton Town Shellfish Hatchery via use of their New York State Department of Environmental Conservation Collector's Permit that gives them permission for such activity. Neither bivalve species investigated here is endangered or protected. Scallops were spawned at the East Hampton Town Shellfish Hatchery located in Montauk, NY, which uses water from Gardiner's Bay, a meso- to oligotrophic, open water body that exchanges with the Atlantic Ocean (pH_NBS_ range: 7.8–8.2, mean  = 8.0±0.14; dissolved oxygen: 210–380 µM, mean  = 280±60 µM; salinity  = 25.9–31.1, mean  = 28.4±1.42; [39]. These pH and dissolved oxygen levels are significantly higher than those found in more eutrophic systems such as the Forge River (Figure 2B) and will be considered ‘normal’ for the purposes of this study. For larval experiments, **e**xperimental vessels (1 L) were filled with 0.2 *µ*m filtered seawater from eastern Shinnecock Bay, New York, USA, and within hours of fertilization, larvae were distributed to each treatment beaker at a concentration of ∼400 individuals L^−1^, consistent with post-spawning densities in estuaries [40]. Larvae were fed an ideal food source, *Isochrysis galbana*, (Tahitian strain, T-Iso) at a rate known to be *ad libitum* (4×10^4^ cells ml^−1^ d^−1^) and to yield maximal bivalve larval growth and survivorship through metamorphosis [32], [33]. Indeed, *I. galbana* densities declined daily demonstrating that larvae were feeding, but never declined by more than 50%. Cultures of *I. galbana* were maintained in exponential phase growth using standard algal culture conditions. To promote high survivorship, containers in contact with larvae were never exposed to chemicals [41]. To discourage the growth of bacteria during experiments, an antibiotic solution (Sigma-Aldrich No. 4083, penicillin, streptomycin, neomycin) was administered to each beaker at 1% its original concentration at the beginning of each experiment and during each water change. This antibiotic mixture at this concentration has been shown to have no negative effects on the growth and survivorship of shellfish larvae [42]. Every three days, larvae were gently poured onto a 64 *µ*m mesh, and condition (live or dead) and developmental stage (veliger, pediveliger, or metamorphosed) of all larvae were recorded. The percent of the population that had metamorphosed at each stage was calculated for surviving individuals only. Dead larvae were identified by a lack of swimming and movement of the velum as well as a loss of pigmentation and/or fully open valves. Surviving larvae from each beaker were transferred into a new vessel with new filtered seawater, food, and antibiotics within a 30 minute period. Experiments lasted 30–40 days post-spawning, thereby covering the early, most critical period in bivalve ontogeny [31], [43], [44]. At the end of the experiments, the diameter of surviving individuals was determined using a dissecting microscope equipped with a Nikon DigiSight Color Digital Camera System (DSVi1) and ImageJ software. Precise CO_2_ levels, dissolved oxygen levels, and complete carbonate chemistry from larval experiments appear in Tables S1 and S2.

### Juvenile experiments

For experiments with juvenile *M. mercenaria*, individuals spawned from eastern Peconic Estuary broodstock (estuarine chemistry as described above) were raised on an algal diet under normal pH and dissolved oxygen conditions (levels as described above) at the East Hampton Town Shellfish Hatchery until experiments were performed. Individuals were transferred to seawater matching the salinity and temperature of the experiment for at least 48 h prior to the start of experiments. *M. mercenaria* of two ages and size classes (two month old  = 6.09±0.65 mm and four month old  = 8.51±0.21 mm) were used for experiments with acidified and low oxygen water achieved via tanked gases. Fifteen individuals of each species were placed into replicate (n = 4), 10 L, high-density polyethylene vessels that were maintained at 20±1°C as described above. Survival of individuals and water chemistry were assessed daily; any dead individuals were removed within <18 h of expiring. Precise CO_2_ levels, dissolved oxygen levels, and complete carbonate chemistry from these experiments appear in Tables S3 and S4. *M. mercenaria* were fed *Isochrysis galbana*, (Tahitian strain, T-Iso) at a density of 4×10^4^ cells ml^−1^ daily. Initial and final shell lengths (distance from the umbo to the ventral edge) of small individuals were determined by means of image capture and digital imaging software (ImageJ), whereas lengths of larger individuals were determined with calipers. Length-based growth rates were calculated by dividing the change in height by the days of the experiment.

### Statistical analyses

Statistical analyses were performed with SigmaStat 4.0 Percent values were arc-sin square root transformed before analyses. Two-way ANOVAs were performed to assess differences among survival, metamorphosis, and growth rates at each CO_2_ and dissolved oxygen level, and were followed by Tukey's multiple comparison tests. One-way ANOVAs were performed to assess the effects of aeration and sodium carbonate on the survival, metamorphosis, growth rates on scallop larvae and were followed by Tukey's multiple comparison tests. Standard or Kruskal-Wallis ANOVAs were performed to assess differences in dissolved oxygen and pH attained in each experiment and were followed by Tukey's multiple comparison tests. Linear regression models of larval scallop survival during experiments were developed to derive daily mortality rates. Values reported in the results sections are means ± one standard deviation.

## Results

**Figure 3:**
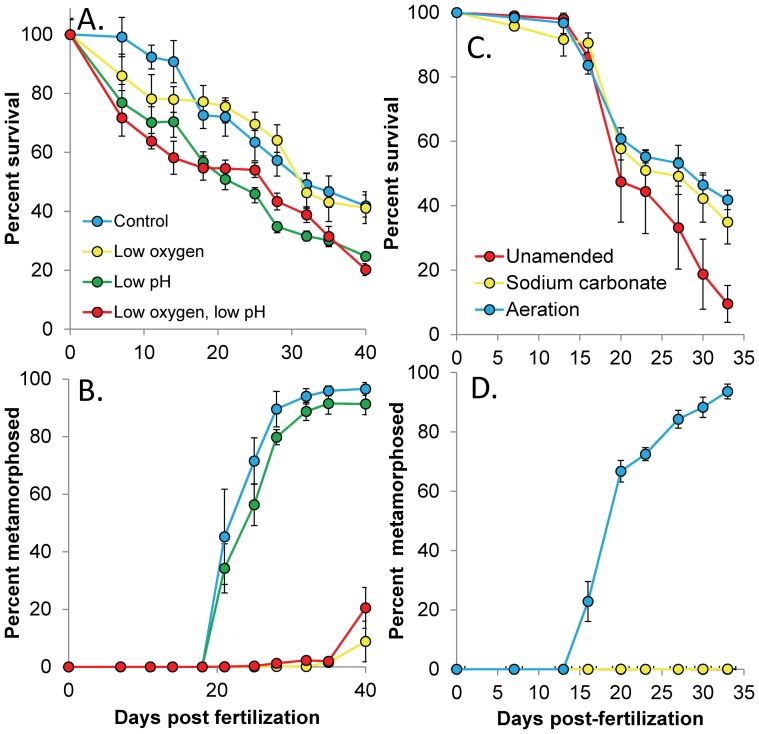
Performance of *A. irradians* larvae exposed to two levels of dissolved oxygen and pH_T_. (A) Percent survival and (B) percent metamorphosis of surviving *A. irradians* larvae during exposure to two levels of pH and dissolved oxygen. (C) Percent survival and (D) percent metamorphosis of surviving *A. irradians* larvae in water naturally low in dissolved oxygen and pH (umamended), low oxygen with normal low pH (via the addition of sodium carbonate), or with normal oxygen and pH (via aeration with and without sodium carbonate). There was no metamorphosis in the unamended and sodium carbonate treatments. Points are means ±1 S.D. Dissolved oxygen and pH levels are presented in Figures 2. Complete carbonate chemistry is given in Tables S1 and S2, while statistical analyses appear in Tables S5, S6, S8, and S9.

**Figure 4:**
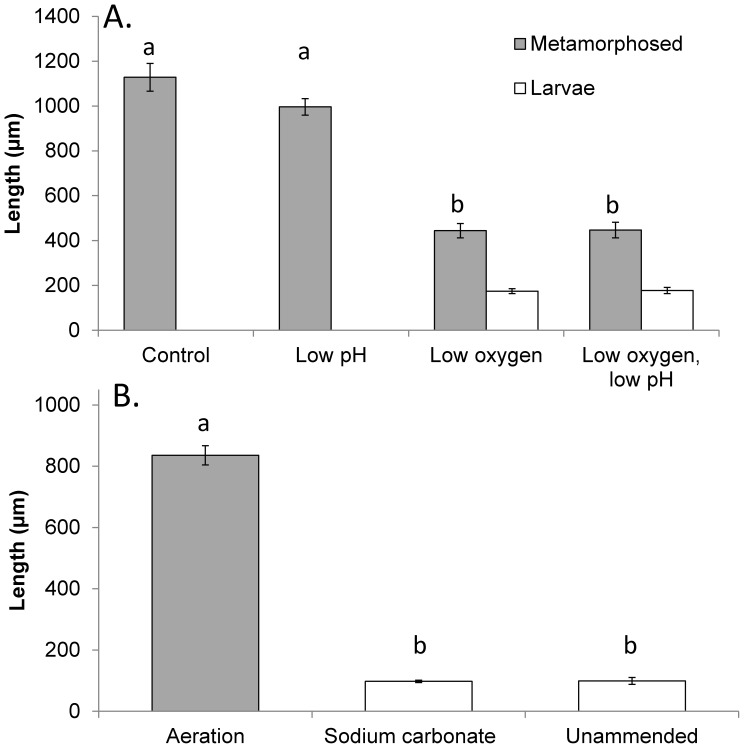
Size of early life stage *A. irradians* larvae, grown under two levels of pH and dissolved oxygen. **A**. Experiment with mixing gases, and B. Experiment manipulating naturally low oxygen and low pH water with aeration and sodium carbonate Bars are means ±S.D. Complete carbonate chemistry from experiments appear in Tables S1 and S2 while statistical analyses appear in Tables S7 and S10. Shared lower case letters indicate treatments that are not significantly different (p>0.05). Statistical comparisons were among metamorphosed individuals only in panel A, but was between individuals of the same cohort that were metamorphosed individuals in the aerated treatment and larvae in other treatments in panel B as no individuals had metamorphosed in those treatments.

In experiments where air, CO_2_, and N_2_ gases were mixed to create hypoxic and acidified conditions for *A. irradians* larvae, levels of dissolved oxygen were significantly lower in the hypoxic and combined low oxygen and acidified treatment (36.5±8.93 µM) relative to the other treatments (260±23.5 µM), while levels of pH were significantly lower in the acidified and combined low oxygen and acidified treatments (pH_T_ = 7.53±0.04) relative to the other treatments (8.00±0.03; *p*<0.05; Figure 2A). These lower pH levels were accompanied by aragonite saturation states (0.79±0.06) that were significantly lower than the control and low oxygen treatments (1.94±0.14; *p*<0.05; Table S1). Under these conditions, larval scallops (*A. irradians*) experienced significantly reduced survival (Figure 3A; *p*<0.05; Table S5) in response to acidification but not hypoxia (Figure 2A), and there was no interaction between the treatments (Table S5). Specifically, daily mortality rates of individuals exposed to normal pH and oxygen, low oxygen, low pH, and low oxygen combined with low pH treatments were 1.5, 1.5, 1.9, and 1.8% per day while total survivorship after 40 d was 42%, 41%, 24%, and 20%, respectively (Figure 3A). In contrast to survival, low oxygen levels significantly reduced the size and delayed metamorphosis of scallop larvae (Figures 3B, 4A; *p*<0.001 for each, respectively Tables S6, S7), while low pH alone did not. The treatments interacted to yield less than additive percentage of metamorphosed larvae while there was no interaction with regard to size (Tables S6, S7). After 25 d, 72% and 56% of surviving individuals had metamorphosed in the control and acidified treatments, while none had metamorphosed in the low oxygen treatments with or without low pH (Figure 3B). After 40 d, 97% and 91% of individuals had metamorphosed in the control and acidified treatments, while only 9% and 2% had done so in the low oxygen treatments with or without low pH (Figure 3B). Metamorphosed individuals from normal oxygen treatments at normal and low pH conditions measured 1,130±16.8 µm and 996±36.7 µm in length, respectively, whereas metamorphosed individuals from low oxygen treatments at normal or low pH conditions were only 445±32.0 and 447±35.0 µm, respectively. Individuals that failed to metamorphose after 40 d in these treatments were even smaller (175±10.9 and 177±14.1 µm; Figure 4A).

Water from Forge River Estuary used for experiments had levels of dissolved oxygen (140±56 µM), pH_T_ (7.37±0.21), carbonate ion (15±9.3 µM), and aragonite saturation (0.23±0.15) that were all significantly lower than the levels produced via the aeration of this water (260±22 µM, 7.91±0.08, 120±24 µM, 1.89±0.15, respectively; *p*<0.05; Figure 2B; Table S2). Aeration did not alter the alkalinity of the water (1848±119 µM for aerated and unamended treatments). The addition of sodium carbonate created water with levels of pH_T_ (7.93±0.08), carbonate ion (98±14 µM), alkalinity (2360±186 µM), and aragonite saturation (1.51±0.24) that were all significantly greater than the unamended Forge River water (*p*<0.05; Figure 2B; Tables S2), but this treatment did not significantly alter levels of dissolved oxygen (142±57 µM; Figure 2B; Tables S2).

Exposure of larval scallops to estuarine waters that were naturally acidified and low in oxygen caused low rates of growth, survival, and metamorphosis, while raising pH or oxygen levels significantly improved different aspects of their performance. For example, while only 10% of larvae survived a 33 d exposure to water with naturally low pH and low oxygen (Figure 2B), the addition of sodium carbonate (pH increase) and aeration (oxygen and pH increase) each significantly increased larval survival ∼ four-fold (to ∼40%; Figure 3C; *p*<0.005; Table S8). During the month long experiment, population mortality rates were 3.2, 2.2, and 2.1% d^−1^ in the unamended, carbonate addition, and aeration treatments, respectively. In contrast, aeration alone significantly increased the size and metamorphic rates of scallop larvae, while sodium carbonate alone did not (Figure 3D, 4B; *p*<0.001; Table S9, S10). Specifically, while >95% of surviving individuals in aerated treatments had metamorphosed after 33 d, 0 and 10% had in the unamended and sodium carbonate treatments (Figure 3D). Similarly, while individuals that were aerated had metamorphosed and grown to 835 µm in length after 33 d, those in the unamended and sodium carbonate treatments had not metamorphosed and were <100 µm (Figure 4B).

**Figure 5:**
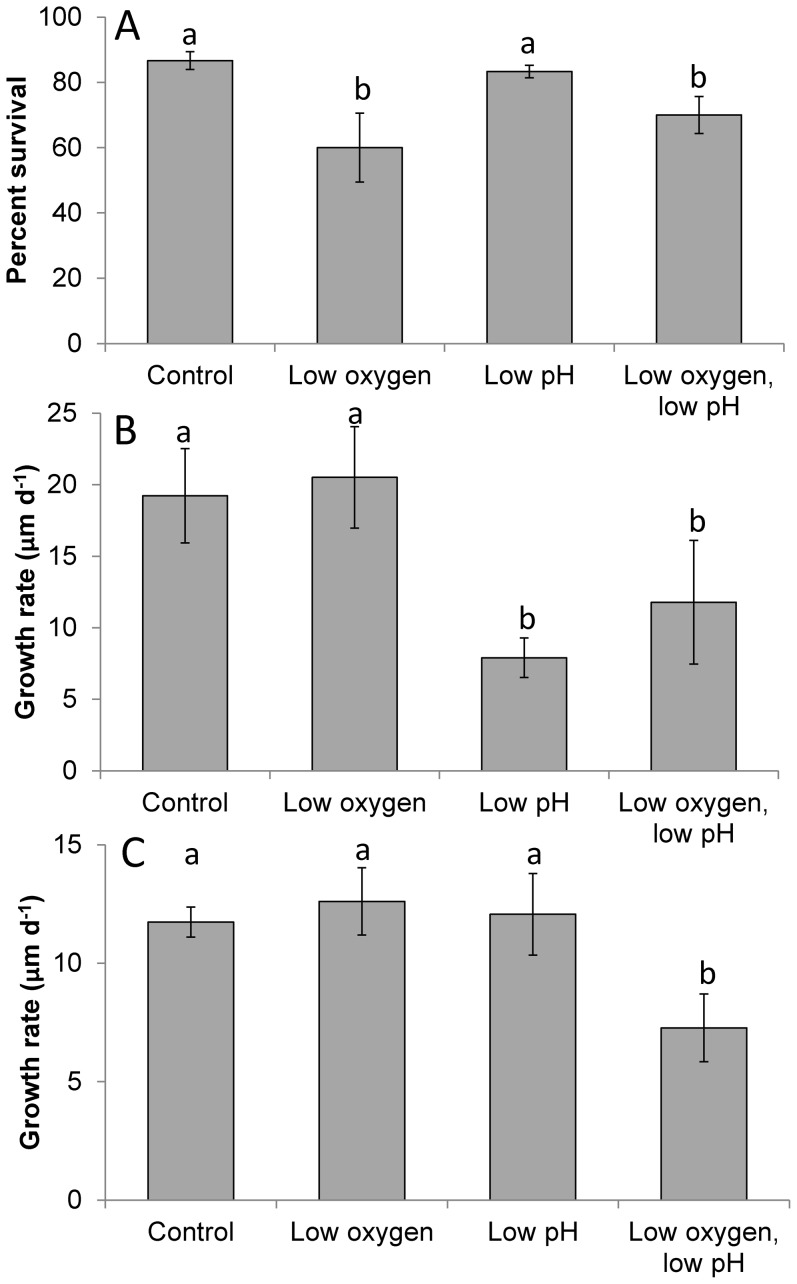
Performance of *M. mercenaria* exposure to two levels of pH and dissolved oxygen. **A.** Percent survival of two month old individuals, **B.** Growth of two month old individuals, **C.** Growth of four month old individuals. Bars are means ±S.D. Dissolved oxygen and pH levels are presented in Figure 2. Complete carbonate chemistry appears in Tables S3 and S4, while statistical analyses appear in Tables S11, S12, and 13. Shared lower case letters indicate treatments that are not significantly different (p>0.05).

Hypoxia and acidification had contrasting and age-dependent effects on juvenile hard clams (*M. mercenaria*). In experiments with early stage juveniles (two-month old), levels of dissolved oxygen were significantly lower in the hypoxic and combined low oxygen and acidified treatment (51.5±6.86 µM) relative to the other treatments (262±6.86 µM), while levels of pH were significantly lower in the acidified and combined low oxygen and acidified treatments (pH_T_ = 7.51±0.01) relative to the other treatments (7.88±0.04; *p*<0.05; Figure 2a). These lower pH levels were accompanied by aragonite saturation states (0.71±0.01) that were significantly lower than the control and low oxygen treatments (1.93±0.07; *p*<0.05; Table S3). Exposure of two-month old juvenile clams to low oxygen significantly reduced their survival (from 87 to 60%; *p*<0.05), while low pH (Figure 2A) did not, and there was no interaction between these treatments (Figure 5A; *p*<0.05, Table S11). In contrast, the low pH water significantly reduced the growth of surviving clams by 60% (from 19 to 7.9 µm d^−1^; Figure 5B; *p*<0.05; Table S12), while low oxygen did not, and there was no interaction between treatments (Table S12).

In experiments with later stage juvenile clams (four months old), levels of dissolved oxygen were significantly lower in the hypoxic and combined low oxygen and acidified treatment (44.3±4.31 µM) relative to the other treatments (289±4.31 µM) while levels of pH were significantly lower in the acidified and combined low oxygen and acidified treatments (pH_T_ = 7.51±0.01) relative to the other treatments (7.96±0.03; *p*<0.05; Figure 2a). These lower pH levels were accompanied by aragonite saturation states (0.70±0.01) that were significantly lower than the control and low oxygen treatments (2.08±0.14; *p*<0.05; Table S4). Four month old clams were more resistant to low oxygen and pH conditions, experiencing high survival in all treatments (>95%). Although their growth rates were not altered by either low oxygen or low pH when administered alone, there was a synergistically negative interaction between these factors as the combined exposure to low oxygen and low pH reduced their growth rates by 40% from 12 µm d^−1^ to 7.3 µm d^−1^ (Figure 5C; *p*<0.05; Table S13).

## Discussion

Regions of the world's oceans with low levels of dissolved oxygen and pH have become increasingly common and will continue to expand in the near future [16], [17], yet the concurrent effects of these two stressors on marine life are largely unknown. Acidification has been shown to inhibit the performance of many calcifying invertebrates [18]–[20] as well as some vertebrates, including fish [45]–[48]. Our findings suggest that the true impact of low oxygen zones on marine life likely requires re-evaluation, as most laboratory studies to date have manipulated oxygen with by administering nitrogen gas [e.g. 21]–[27] resulting in concurrent basification rather than acidification (Figure 1). Given that this approach misrepresents natural marine ecosystems (Figure 1), it may mask the true effects of low oxygen zones on marine animals. For example, while low oxygen severely delayed the metamorphosis and greatly restricted the growth of larval scallops, their survival was only compromised when they were concurrently exposed to the acidified conditions. While juvenile clams responded differently to hypoxia and acidification, the full effects of these processes could not have been predicted by considering only one of these treatments alone. For example, while early stage juvenile clams suffered enhanced mortality under hypoxic conditions, their growth rates were depressed by acidification. More surprising was the synergistically negative effect of concurrent acidification and hypoxia on the growth rates of late stage clams, which was not detected in either individual treatment. While the two species of bivalves studied here have been shown to be differentially susceptible to acidification [19], the present study demonstrates that they also respond differentially to hypoxia and acidification. As such, it is likely that making broad predictions regarding the response of ocean animals to these stressors will prove challenging.

Prior ecosystem studies investigating the distribution of coastal marine organisms within low oxygen zones have often not measured pH or carbonate chemistry and have interpreted findings solely through the lens of oxygen concentrations [e.g. 49]–[52]. By not concurrently considering acidification within these systems, the processes driving distribution patterns may be misinterpreted. In contrast, a recent open ocean study of oxygen minimum zones (OMZs) found that pteropods migrating into OMZs are more resistant to acidification than those that do not [53], suggesting that acidification may be as important as oxygen in shaping the distribution of these organisms within and around these zones.

Recent research has robustly demonstrated the sensitive nature of early life stage bivalves to the levels of ocean acidification projected for the end of this century [19], [20]. Concurrently, it has been established that some eutrophic coastal waters are already experiencing seasonal acidification [10]–[14]. Our novel experiments with water naturally low oxygen and low pH demonstrate that these conditions negatively impact larval shellfish. For example, consistent with our experiments using mixed gases, increasing the pH of acidified coastal waters with sodium carbonate significantly improved larval survival, thereby suggesting that contemporary acidification in eutrophic coastal zones may already be reducing the survival of bivalve larvae and perhaps other organisms. Water used for experiments had levels of pH and pCO_2_ (7.36±0.21 and 2620±781 µatm, respectively; Table S2) that are projected to occur in open ocean surface waters under ‘business-as-usual’ scenarios in hundreds of years [54], [55]. As such, eutrophic coastal zones may be relatively less impacted by future atmospheric CO_2_ driven acidification than open ocean environments [56] but may also provide early insight into the manner in which future climate change may shape the distribution and abundance of ocean animals [14], [57], [58]. Interestingly, mean oxygen levels during our experiment with eutrophic coastal water were low (<140 µM) but higher than levels achieved during our mixed gas experiments, yet they still reduced the size and delayed the metamorphosis of larval scallops. Given that larval bivalves with extended metamorphosis times or of smaller sizes are susceptible to higher cumulative predation rates [59], [60], mortality rates of larvae exposed to low oxygen conditions in the wild may be even greater than those observed during our experiments. Such elevated mortality among early life stages of bivalves would be expected to influence the distribution and abundance of adult populations [31], [44]. While ammonium in the Forge River was not at levels known to be toxic (<25 µM), additional stressors present in hypoxic waters such as sulfides may have further inhibited the performance of scallop larvae. Hence, future research regarding the effects of hypoxic zones on marine life may need to consider stressors beyond oxygen and pH such as elevated sulfides, ammonium, and temperature [3], [61], [62].

Our findings coupled with prior studies provide insight regarding the differing physiological mechanisms by which hypoxia and acidification may impact early life stage bivalves. Marine invertebrates commonly adapt to environmental stressors by reducing their metabolic rate, and over an extended period of time this can translate in lowered growth rates and smaller sizes [63], [64]. Accordingly, acidification and hypoxia both contributed toward smaller individuals in the experiments presented here. In a manner consistent with prior studies [21], [22], it was exposure of larval stages to low oxygen, however, that had the largest negative effect on the growth of individuals. Larvae also experienced delayed metamorphosis suggesting these processes are linked and that the lower metabolic state induced by hypoxia does not afford larvae the aerobic scope to initiate metamorphosis. Interestingly, larvae exposed to hypoxia (but not anoxia) commonly do not suffer enhanced mortality [21,22, this study], suggesting that the physiological response of reduced metabolism, growth, and metamorphosis are sufficient for sustaining individuals during extended hypoxia, at least during the larval stage.

Regarding acidification, research has demonstrated that lowered pH and the associated reduction in carbonate ion availability can impact multiple physiological pathways in bivalves beginning with calcification [65], [66]. Lowered rates of calcification can have cascading effects on larval physiology, inhibiting other biochemical processes such as RNA synthesis and lipid content [19], [66] as well as more general indicators of metabolism such as growth and size [20], [40]. For some species of bivalve larvae, including *A. irradians* examined here, exposure to undersaturated levels of calcium carbonate during their first week of development can elicit depressed rates of survival [19], [66]–[68]. Consistent with this finding, the largest differences between survival in acidification and normal pH treatments were observed during exposure of larval stage bivalve to acidification. Hence, simply lowering metabolic rates may not be a sufficient physiological response to overcome the compromised calcification associated with acidification in the early stages of some larval bivalves.

There may be a physiological feedback and synergy between low oxygen and low pH in marine invertebrates [63]. As environmental acidification and low oxygen slow metabolic rates, internal gas exchange may slow, intracellular oxygen levels may be reduced, and CO_2_ from respiration may accumulate, potentially exacerbating hypercapnia and thus further lowering metabolism. This is consistent with our finding that the combination of low oxygen and low pH, but not either factor alone, inhibited the growth of the later stage hard clams. Finally, our results suggest that, in a manner consistent with the age-dependent effects of ocean acidification on early life stage bivalves [69], the effects of hypoxia and acidification on early life stage bivalves are also age dependent. While larvae exposed to both stressors experienced, up to 75% reduction in survival, early stage juvenile survival was more modestly reduced by the two stressors (20%). The two stressors combined only reduced growth (by 20%) not survival, in later stage juveniles.

Hypoxia and acidification are increasingly co-occurring in the ocean [10,11,14, this study], and understanding the effects of such climate change stressors on marine animals is a significant challenge. In low oxygen regions of the ocean, the tissue demand for oxygen of marine animals is often not met by ambient supply, and thus the ability of these organisms to tolerate secondary stressors such as acidification could be compromised [63], [64]. Thus, it is possible that even animals insensitive to acidification alone could be negatively affected by low pH when concurrently exposed to hypoxic conditions. Given that fish are generally more vulnerable to low oxygen than mollusks such as the bivalves studied here [4] and that some early life stage fish have already been shown to be sensitive to acidification [45]–[47], the dual effects of hypoxia and acidification on marine teleosts may be profound.

Because hypoxia threatens fisheries and biodiversity in coastal ecosystems [2], [4], environmental regulatory agencies often set management goals for dissolved oxygen to protect estuarine animals based on levels known to negatively affect marine life (e.g., 2 mg L^−1^ O_2_).[4], [70]–[72]. Given our finding that concurrent acidification and low oxygen significantly and independently depressed growth and survival rates of estuarine bivalves, it is plausible that coastal managerial criteria based strictly on oxygen levels, but not pH, may not adequately protect marine life in some ecosystems. Therefore, future environmental regulations developed to protect estuarine organisms in regions prone to hypoxia should consider the concurrent effects of acidification on these animals, particularly as climate change accelerates the intensity of acidification in coastal zones.

The expansion of hypoxic zones in coastal and open oceans is one of many predicted consequences of intensifying global climate change [9], [15], [17]. While microbial processes will continue to promote acidification in these regions [11], this will be exacerbated by the loading of anthropogenic CO_2_ [16]–[18]. We suggest that recently discovered high CO_2_ sensitivities in many finfish and shellfish [20], [45]–[47], and the compounded effects of high CO_2_ and low O_2_ in bivalves relative to each individual parameter should prompt a re-alignment of future studies. A comprehensive evaluation of the combined effects of low oxygen and acidification on marine life is critical for understanding how ocean ecosystems respond to these conditions both today and under future climate change scenarios.

## Supporting Information

Table S1 **Mean temperature, pH, dissolved oxygen, carbonate chemistry, alkalinity, and salinity (±1 SD) during the experiment exposing larval stage** ***Argopecten irradians*** **to differing levels of pH and dissolved oxygen achieved via mixing tanked gases.**
(DOC)

Table S2 **Mean temperature, pH, dissolved oxygen, carbonate chemistry, alkalinity, and salinity (±1 SD) during the experiment exposing larval stage** ***Argopecten irradians*** **to differing levels of pH and dissolved oxygen achieved via the addition of aeration and sodium carbonate to estuarine water with naturally low oxygen and low pH.**
(DOC)

Table S3 **Mean temperature, pH, dissolved oxygen, carbonate chemistry, alkalinity, and salinity (±1 SD) during the experiment exposing two month old** ***Mercenaria mercenaria*** **to differing levels of pH and dissolved oxygen achieved via mixing tanked gases.**
(DOC)

Table S4 **Mean temperature, pH, dissolved oxygen, carbonate chemistry, alkalinity, and salinity (±1 SD) during the experiment exposing four month old** ***Mercenaria mercenaria*** **to differing levels of pH and dissolved oxygen achieved via mixing tanked gases.**
(DOC)

Table S5 **Two-way analysis of variance for** ***Argopecten irradians*** **larval survival when exposed to two levels of dissolved oxygen and pH.**
(DOC)

Table S6 **Two-way analysis of variance for** ***Argopecten irradians*** **larval metamorphosis when exposed to two levels of dissolved oxygen and pH.**
(DOC)

Table S7 **Two-way analysis of variance for** ***Argopecten irradians*** **larval size when exposed to two levels of dissolved oxygen and pH.**
(DOC)

Table S8 **One-way analysis of variance for** ***Argopecten irradians*** **larval survival when exposed to hypoxic and acidified Forge River water amended with sodium carbonate or aeration.**
(DOC)

Table S9 **One-way analysis of variance for** ***Argopecten irradians*** **larval metamorphosis when exposed to hypoxic and acidified Forge River water amended with sodium carbonate or aeration.**
(DOC)

Table S10 **One-way analysis of variance for** ***Argopecten irradians*** **larval size when exposed to hypoxic and acidified Forge River water amended with sodium carbonate or aeration.**
(DOC)

Table S11 **Two-way analysis of variance for survival of two-month old** ***Mercenaria mercenaria*** **exposed to two levels of dissolved oxygen and pH.**
(DOC)

Table S12 **Two-way analysis of variance for growth rates of two-month old** ***Mercenaria mercenaria*** **exposed to two levels of dissolved oxygen and pH.**
(DOC)

Table S13 **Two-way analysis of variance for growth rates of four-month old** ***Mercenaria mercenaria*** **exposed to two levels of dissolved oxygen and pH.**
(DOC)
