# Supplementary material for: Hypoxia and Acidification Have Additive and Synergistic Negative Effects on the Growth, Survival, and Metamorphosis of Early Life Stage Bivalves
Source: PLoS One. 2014 Jan 8;9(1):e83648. doi: 10.1371/journal.pone.0083648 (PMC3885513; doi:10.1371/journal.pone.0083648)
Supplement: Table S2 — Mean temperature, pH, dissolved oxygen, carbonate chemistry, alkalinity, and salinity (±1 SD) during the experiment exposing larval stage Argopecten irradians to differing levels of pH and dissolved oxygen achieved via the addition of aeration and sodium carbonate to estuarine water with naturally low oxygen and low pH. (DOC) [file pone.0083648.s002.doc]

**Table S2**. Mean temperature, pH, dissolved oxygen, carbonate chemistry, alkalinity, and salinity (± 1 SD) during the experiment exposing larval stage *Argopecten irradians* to differing levels of pH and dissolved oxygen achieved via the addition of aeration and sodium carbonate to estuarine water with low oxygen and low pH.
