# Supplementary material for: Hypoxia and Acidification Have Additive and Synergistic Negative Effects on the Growth, Survival, and Metamorphosis of Early Life Stage Bivalves
Source: PLoS One. 2014 Jan 8;9(1):e83648. doi: 10.1371/journal.pone.0083648 (PMC3885513; doi:10.1371/journal.pone.0083648)
Supplement: Table S4 — Mean temperature, pH, dissolved oxygen, carbonate chemistry, alkalinity, and salinity (±1 SD) during the experiment exposing four month old Mercenaria mercenaria to differing levels of pH and dissolved oxygen achieved via mixing tanked gases. (DOC) [file pone.0083648.s004.doc]

**Table S4**. Mean temperature, pH, dissolved oxygen, carbonate chemistry, alkalinity, and salinity (± 1 SD) during the experiment exposing four month old *Mercenaria mercenaria* to differing levels of pH and dissolved oxygen achieved via mixing tanked gases.
