# Supplementary material for: Hypoxia and Acidification Have Additive and Synergistic Negative Effects on the Growth, Survival, and Metamorphosis of Early Life Stage Bivalves
Source: PLoS One. 2014 Jan 8;9(1):e83648. doi: 10.1371/journal.pone.0083648 (PMC3885513; doi:10.1371/journal.pone.0083648)
Supplement: Table S5 — Two-way analysis of variance for Argopecten irradians larval survival when exposed to two levels of dissolved oxygen and pH. (DOC) [file pone.0083648.s005.doc]

**Table S5**. Two-way analysis of variance for *Argopecten irradians* larval survival when exposed to two levels of dissolved oxygen and pH.

| Source of variation | *df* | *SS* | *MS* | *F-ratio* | *p-value* |
| --- | --- | --- | --- | --- | --- |
| Dissolved oxygen | 1 | 0.00393 | 0.00393 | 0.741 | 0.406 |
| pH | 1 | 0.169 | 0.169 | 31.959 | <0.001 |
| Dissolved oxygen & pH | 1 | 0.00222 | 0.00222 | 0.418 | 0.53 |
| Residual | 12 | 0.0636 | 0.0053 |  |  |
| Total | 15 | 0.239 | 0.0159 |  |  |
|  |  |  |  |  |  |
