# Supplementary material for: Hypoxia and Acidification Have Additive and Synergistic Negative Effects on the Growth, Survival, and Metamorphosis of Early Life Stage Bivalves
Source: PLoS One. 2014 Jan 8;9(1):e83648. doi: 10.1371/journal.pone.0083648 (PMC3885513; doi:10.1371/journal.pone.0083648)
Supplement: Table S6 — Two-way analysis of variance for Argopecten irradians larval metamorphosis when exposed to two levels of dissolved oxygen and pH. (DOC) [file pone.0083648.s006.doc]

**Table S6**. Two-way analysis of variance for *Argopecten irradians* larval metamorphosis when exposed to two levels of dissolved oxygen and pH.

| Source of variation | *df* | *SS* | *MS* | *F-ratio* | *p-value* |
| --- | --- | --- | --- | --- | --- |
| Dissolved oxygen | 1 | 1.611 | 1.611 | 80.317 | <0.001 |
| pH | 1 | 0.0364 | 0.0364 | 1.813 | 0.203 |
| Dissolved oxygen & pH | 1 | 0.3 | 0.3 | 14.949 | 0.002 |
| Residual | 12 | 0.241 | 0.0201 |  |  |
| Total | 15 | 2.188 | 0.146 |  |  |
