# Supplementary material for: Hypoxia and Acidification Have Additive and Synergistic Negative Effects on the Growth, Survival, and Metamorphosis of Early Life Stage Bivalves
Source: PLoS One. 2014 Jan 8;9(1):e83648. doi: 10.1371/journal.pone.0083648 (PMC3885513; doi:10.1371/journal.pone.0083648)
Supplement: Table S7 — Two-way analysis of variance for Argopecten irradians larval size when exposed to two levels of dissolved oxygen and pH. (DOC) [file pone.0083648.s007.doc]

**Table S7**. Two-way analysis of variance for *Argopecten irradians* larval size when exposed to two levels of dissolved oxygen and pH.

| Source of variation | *df* | *SS* | *MS* | *F-ratio* | *p-value* |
| --- | --- | --- | --- | --- | --- |
| Dissolved oxygen | 1 | 127821.3 | 127821.3 | 644.912 | <0.001 |
| pH | 1 | 301.81 | 301.81 | 1.523 | 0.241 |
| Dissolved oxygen & pH | 1 | 734.9 | 734.9 | 3.708 | 0.078 |
| Residual | 12 | 2378.397 | 198.2 |  |  |
| Total | 15 | 131236.4 | 8749.094 |  |  |
