# Supplementary material for: Hypoxia and Acidification Have Additive and Synergistic Negative Effects on the Growth, Survival, and Metamorphosis of Early Life Stage Bivalves
Source: PLoS One. 2014 Jan 8;9(1):e83648. doi: 10.1371/journal.pone.0083648 (PMC3885513; doi:10.1371/journal.pone.0083648)
Supplement: Table S8 — One-way analysis of variance for Argopecten irradians larval survival when exposed to hypoxic and acidified Forge River water amended with sodium carbonate or aeration. (DOC) [file pone.0083648.s008.doc]

**Table S8**. One-way analysis of variance for *Argopecten irradians* larval survival when exposed to hypoxic and acidified Forge River water amended with sodium carbonate or aeration.

| Source of variation | *df* | *SS* | *MS* | *F-ratio* | *p-value* |
| --- | --- | --- | --- | --- | --- |
| Between Groups | 2 | 0.408 | 0.204 | 10.785 | 0.004 |
| Residual | 9 | 0.17 | 0.0189 |  |  |
| Total | 11 | 0.579 |  |  |  |
