# Supplementary material for: Hypoxia and Acidification Have Additive and Synergistic Negative Effects on the Growth, Survival, and Metamorphosis of Early Life Stage Bivalves
Source: PLoS One. 2014 Jan 8;9(1):e83648. doi: 10.1371/journal.pone.0083648 (PMC3885513; doi:10.1371/journal.pone.0083648)
Supplement: Table S10 — One-way analysis of variance for Argopecten irradians larval size when exposed to hypoxic and acidified Forge River water amended with sodium carbonate or aeration. (DOC) [file pone.0083648.s010.doc]

**Table S10**. One-way analysis of variance for *Argopecten irradians* larval size when exposed to hypoxic and acidified Forge River water amended with sodium carbonate or aeration.

| Source of variation | *df* | *SS* | *MS* | *F-ratio* | *p-value* |
| --- | --- | --- | --- | --- | --- |
| Between Groups | 2 | 1424508 | 712254 | 2524.712 | <0.001 |
| Residual | 9 | 2539.01 | 282.113 |  |  |
| Total | 11 | 1427047 |  |  |  |
