# Supplementary material for: Hypoxia and Acidification Have Additive and Synergistic Negative Effects on the Growth, Survival, and Metamorphosis of Early Life Stage Bivalves
Source: PLoS One. 2014 Jan 8;9(1):e83648. doi: 10.1371/journal.pone.0083648 (PMC3885513; doi:10.1371/journal.pone.0083648)
Supplement: Table S11 — Two-way analysis of variance for survival of two-month old Mercenaria mercenaria exposed to two levels of dissolved oxygen and pH. (DOC) [file pone.0083648.s011.doc]

**Table S11**. Two-way analysis of variance for survival of two-month old *Mercenaria mercenaria* exposed to two levels of dissolved oxygen and pH.

| Source of variation | *df* | *SS* | *MS* | *F-ratio* | *p-value* |
| --- | --- | --- | --- | --- | --- |
| Dissolved oxygen | 1 | 0.162 | 0.162 | 8.106 | 0.015 |
| pH | 1 | 0.0114 | 0.0114 | 0.569 | 0.465 |
| Dissolved oxygen & pH | 1 | 0.0432 | 0.0432 | 2.159 | 0.167 |
| Residual | 12 | 0.24 | 0.02 |  |  |
| Total | 15 | 0.457 | 0.0305 |  |  |
