# Supplementary material for: Hypoxia and Acidification Have Additive and Synergistic Negative Effects on the Growth, Survival, and Metamorphosis of Early Life Stage Bivalves
Source: PLoS One. 2014 Jan 8;9(1):e83648. doi: 10.1371/journal.pone.0083648 (PMC3885513; doi:10.1371/journal.pone.0083648)
Supplement: Table S12 — Two-way analysis of variance for growth rates of two-month old Mercenaria mercenaria exposed to two levels of dissolved oxygen and pH. (DOC) [file pone.0083648.s012.doc]

**Table S12**. Two-way analysis of variance for growth rates of two-month old *Mercenaria mercenaria* exposed to two levels of dissolved oxygen and pH.

| Source of variation | *df* | *SS* | *MS* | *F-ratio* | *p-value* |
| --- | --- | --- | --- | --- | --- |
| Dissolved oxygen | 1 | 6.26E-05 | 6.26E-05 | 1.518 | 0.242 |
| pH | 1 | 0.000272 | 0.000272 | 6.602 | 0.025 |
| Dissolved oxygen & pH | 1 | 2.93E-05 | 2.93E-05 | 0.71 | 0.416 |
| Residual | 12 | 0.000495 | 4.12E-05 |  |  |
| Total | 15 | 0.000859 | 5.72E-05 |  |  |
