# Supplementary material for: Hypoxia and Acidification Have Additive and Synergistic Negative Effects on the Growth, Survival, and Metamorphosis of Early Life Stage Bivalves
Source: PLoS One. 2014 Jan 8;9(1):e83648. doi: 10.1371/journal.pone.0083648 (PMC3885513; doi:10.1371/journal.pone.0083648)
Supplement: Table S13 — Two-way analysis of variance for growth rates of four-month old Mercenaria mercenaria exposed to two levels of dissolved oxygen and pH. (DOC) [file pone.0083648.s013.doc]

**Table S13**. Two-way analysis of variance for growth rates of four-month old *Mercenaria mercenaria* exposed to two levels of dissolved oxygen and pH.

| Source of variation | *df* | *SS* | *MS* | *F-ratio* | *p-value* |
| --- | --- | --- | --- | --- | --- |
| Dissolved oxygen | 1 | 1.14E-05 | 1.14E-05 | 1.536 | 0.239 |
| pH | 1 | 0.00002 | 0.00002 | 2.688 | 0.127 |
| Dissolved oxygen & pH | 1 | 3.85E-05 | 3.85E-05 | 5.188 | 0.042 |
| Residual | 12 | 8.91E-05 | 7.43E-06 |  |  |
| Total | 15 | 0.000159 | 1.06E-05 |  |  |
